# Laser-sound: optoacoustic transduction from digital audio streams

**DOI:** 10.1038/s41598-020-78990-z

**Published:** 2021-01-22

**Authors:** Konstantinos Kaleris, Björn Stelzner, Panagiotis Hatziantoniou, Dimosthenis Trimis, John Mourjopoulos

**Affiliations:** 1 Department of Electrical and Computer Engineering, Wire Communications Laboratory, Audio and Acoustic Technology Group, University of Patras, 26500 Rio, Greece; 2 Karlsruhe Institute of Technology, Engler-Bunte-Institute, Karlsruhe, Germany

**Keywords:** Photoacoustics, Engineering

## Abstract

This work presents a novel laser-based optoacoustic transducer capable of reproducing controlled and continuous sound of arbitrary complexity in the air or on solid targets. Light-to-sound transduction is achieved via laser-induced breakdown, leading to the formation of plasma acoustic sources in any desired spatial location. The acoustic signal is encoded into pulse streams via a discrete-time audio modulation and is reproduced by fast consecutive excitation of the target medium with appropriately modulated laser pulses. This results in the signal being directly reconstructed at the desired location of the target medium without the need for a receiver or demodulation device. In this work, the principles and evaluation results of such a novel laser-sound prototype system are presented. The performance of the prototype is evaluated by systematic experimental measurements of audio test signals, from which the basic acoustical response is derived. Moreover, a generic computational model is presented that allows for the simulation of laser-sound reproduction of 1-bit or multibit audio streams. The model evaluations are validated by comparison with the acoustic measurements, whereby a good agreement is found. Finally, the computational model is used to simulate an ideal optoacoustic transducer based on the specifications of state-of-the-art commercially available lasers.

## Introduction

Sound reproduction in the audible frequency range relies on transducer technology that was established more than 100 years ago. The technology is mainly based on the electromagnetic loudspeakers^1^, since alternative implementations, such as electrostatic^2^, piezoelectric^3^, thermoacoustic^4,5^ and voltage-driven plasma loudspeakers^6^, do not constitute viable solutions for most audio reproduction applications. However, the acoustic power, directivity, response, efficiency and controllability of the traditional electroacoustic loudspeakers are significantly limited, mainly due to: (a) the weak coupling of the moving-coil electromechanical driver to the low density air medium, (b) the inability to digitally control and directly transcode audio data into acoustic waves, (c) the requirement for local electric power supply to drive low-efficiency transducer units, (d) the need for multiple and distant transducers to achieve controlled spatial reproduction from distributed sound sources.

**Figure 1:**
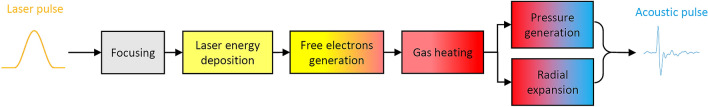
Conceptual diagram of the physical processes behind laser-induced optoacoustic transduction.

Over the last decades, laser-driven sound and ultrasound generation has attracted interest in a variety of scientific fields and applications, such as laser-induced breakdown spectroscopy^7,8^ (LIBS), inertial nuclear fusion^9^, control of meteorological phenomena^10,11^, military and defense applications^12,13^ and others. Laser-driven sound generation takes place when short or ultrashort laser pulses of sufficient energy are focused into a gaseous, liquid or solid target. The interaction of the intense light radiation with the targeted material leads to the generation of free electrons and to the formation of a plasma volume. Particularly in gases and liquids, the plasma formation is followed by a rapid thermoelastic expansion and successive collapse of the ionized volume, leading to the emission of an acoustic pulse^14,15^. Figure 1 shows a conceptual diagram of the physical processes behind laser-induced breakdown (LIB) in gases. Similarly, when sufficiently intense laser pulses are focused on a solid target, the ablated fragments of the material push the surrounding air molecules generating an acoustic wave^16,17^. These processes enable the generation of massless sound sources, which can potentially be formed at arbitrary positions and distances from the driving laser unit with high localization precision. Moreover, modulated trains of laser pulses emitted by appropriate triggering of the laser can carry complex sound information and the optical power required for the transduction to strong acoustic waves at the desired position in space. Laser-generated sound waves can be very rapid and strong, while they enable the rendering of moving virtual sound sources in space by real-time shifting of the laser focal spot within the possible beam-focusing area. The generated acoustic pulse trains enable the direct reproduction of digital sound streams without the need for digital-to-analog conversion, allowing for all-digital audio reproduction chains. In addition, laser-generated sound sources do not involve moving parts and as demonstrated in the next section, they exhibit well-defined and stable frequency response which is not subject to constructional or material defects (see also Discussion section).

Laser-generated acoustic pulses have been studied experimentally and analytically in numerous works^18–22^. Ιt is well known that they exhibit an N-wave signature in the time domain, while their frequency spectra extend from the low “subwoofer” audible frequencies up to the ultrasounds^18,22,23^. For optical intensities well above the breakdown threshold, the time–frequency characteristics of the acoustic pulses are highly repeatable, depending only on the parameters of the optical radiation, such as pulse wavelength, duration, energy, focusing conditions and on the properties of the target material. As a result, these can be controlled by appropriate modulation of the laser radiation. These characteristics, along with their sufficient acoustic energy and peak pressure, render the laser-generated N-pulses suitable for acoustics applications and several have been proposed in the last few years. Bolanos et al. have shown that laser-generated N-pulses are suitable for the acoustic evaluation of auditory spaces and acoustic scale models^24,25^. Eskelinen et al. have proposed the generation of directional and steerable acoustic sources via the fast excitation of multiple laser-plasma sources in air^26^. Recently, Kaleris et al. have demonstrated the possibility to exploit laser filamentation for the formation of acoustic sources with controlled directivity in air^23^. However, all of these works focus on discrete laser-generated N-waves.

Although no implementation or model for the laser-driven reproduction of complex and continuous sound has been presented so far, such a technology is practically feasible. The pulsed nature of the laser-generated acoustic waves indicates the need for a digital audio modulation to transcode complex sound signals, in analogy to the well-established pulse streams used by digital audio systems. Pulse modulations such as Pulse Width Modulation^27–29^ (PWM) and Sigma-Delta Modulation^30,31^ (SDM or ΣΔ modulation) are extensively used in contemporary audio devices^32,33^, for example in audio amplifiers^34,35^, embedded audio processors and Digital to Analog converters^30^. Particularly, ΣΔ modulation is widely used in analog-to-digital (ADC) and digital-to-analog (DAC) conversion, transmission and storage of audio data^32,33^. In the ΣΔ modulation, the analog or digital input audio signal is converted into a stream of rectangular pulses or a bit stream, when considering an analog or digital modulator output, respectively. The encoding can be either 1-bit or k-bit, the latter corresponding to pulses with ${2}^{k}-1$ possible amplitudes. The information of the modulating audio signal, particularly the changes in its amplitude, is encoded in the ΣΔ signal as a pulse density that varies with time. ΣΔ modulation requires oversampling to distribute the quantization noise across a wide frequency range, which extends well beyond the frequency range of the input signal, hereafter referred to as inband range or band of interest. Additionally, a high-pass spectral profile of the quantization noise is achieved via noise shaping^30^, resulting in a further reduction of the quantization noise energy within the band of interest. The noise shaping profile depends on the order of the modulator, where a higher order corresponds to a reduced total inband noise energy and a steeper slope of the high-pass profile (see ΣΔ modulator structure subsection).

In this work, we present a novel optoacoustic transducer prototype that is capable of reproducing arbitrarily complex, continuous and controlled sound signals via laser-induced breakdown directly in the air, driven by digital audio streams. The transducer is based on a single laser unit that can emit high-energy laser pulses at high repetition rates, allowing for fast consecutive optical excitation of the target medium that leads to the formation of highly dense acoustic pulse streams. The system is driven by digital audio data based on ΣΔ modulation, which enables the encoding of complex audio information into streams of laser-generated N-pulses. The adoption of the ΣΔ modulation emerges naturally from the physical characteristics and requirements of the laser-sound system. From a physics point of view, the N-shaped acoustic pulses generated by gas breakdown have a duration of several tens of microseconds^21,22,24^, allowing for the formation of acoustic pulse streams with real-time controllable and sufficiently high pulse repetition rates. It is noted that for the typical single-bit ΣΔ modulation used in audio applications, i.e. the Direct Stream Digital (DSD)^33^, a 2.8224 MHz sampling frequency is adopted that corresponds to a minimum pulse-to-pulse time interval of ~ 0.35 μs. These requirements are far beyond the capabilities of currently available state-of-the-art lasers. However, multibit ΣΔ modulation schemes that are commonly used in ΣΔ audio DACs^36^ can be adopted also for laser-driven sound generation. Using such a modulation, the required repetition rate is significantly reduced, as shown in the Simulation of laser-driven audio reproduction subsection. Similarly, from the signal’s point of view, although the frequency spectrum of such laser-generated ΣΔ streams extends well beyond the frequency range of the input signal, the spectral content of the input signal is preserved in the modulated signal in the band of interest. Consequently, the N-pulse stream can be reproduced directly in the air or on solid targets without the need for a receiver or demodulation device^34^.

Here, the performance of the optoacoustic transducer is evaluated via systematic acoustic measurements of reproduced test audio signals, particularly sine waves, sine sweeps, speech and music signals. In addition, a mathematical model is presented for the simulation of laser-generated digital audio streams of arbitrary complexity and their demodulation within the audible frequency range. The model’s predictions are validated by comparison to measured signals reproduced by the transducer prototype. The mathematical model constitutes a complete tool for the simulation and design of laser-sound systems, as it enables the evaluation of the acoustic response of any ΣΔ-based laser-sound system in the time and frequency domain with high precision.

This paper is structured as follows. In the Evaluation of the laser-sound optoacoustic transducer section, the computational model for the simulation of the laser-generated pulse streams is developed. Moreover, the results from the systematic measurements of the acoustic signals reproduced by the laser-sound prototype system are presented and compared to the simulations. The model is also used to simulate an ideal laser-based optoacoustic transducer in the audible range, as this exceeds the technical capabilities of the prototype system. The Discussion section summarizes the findings of this work and lays the foundations of an all-digital laser-driven audio system capable of reproducing high-fidelity sound within the audible spectrum via massless, spatially unbound acoustic sources. Finally, the Methods section provides details on the experimental setup and the deployed signal processing techniques used.

## Evaluation of the laser-sound optoacoustic transducer

**Figure 2:**
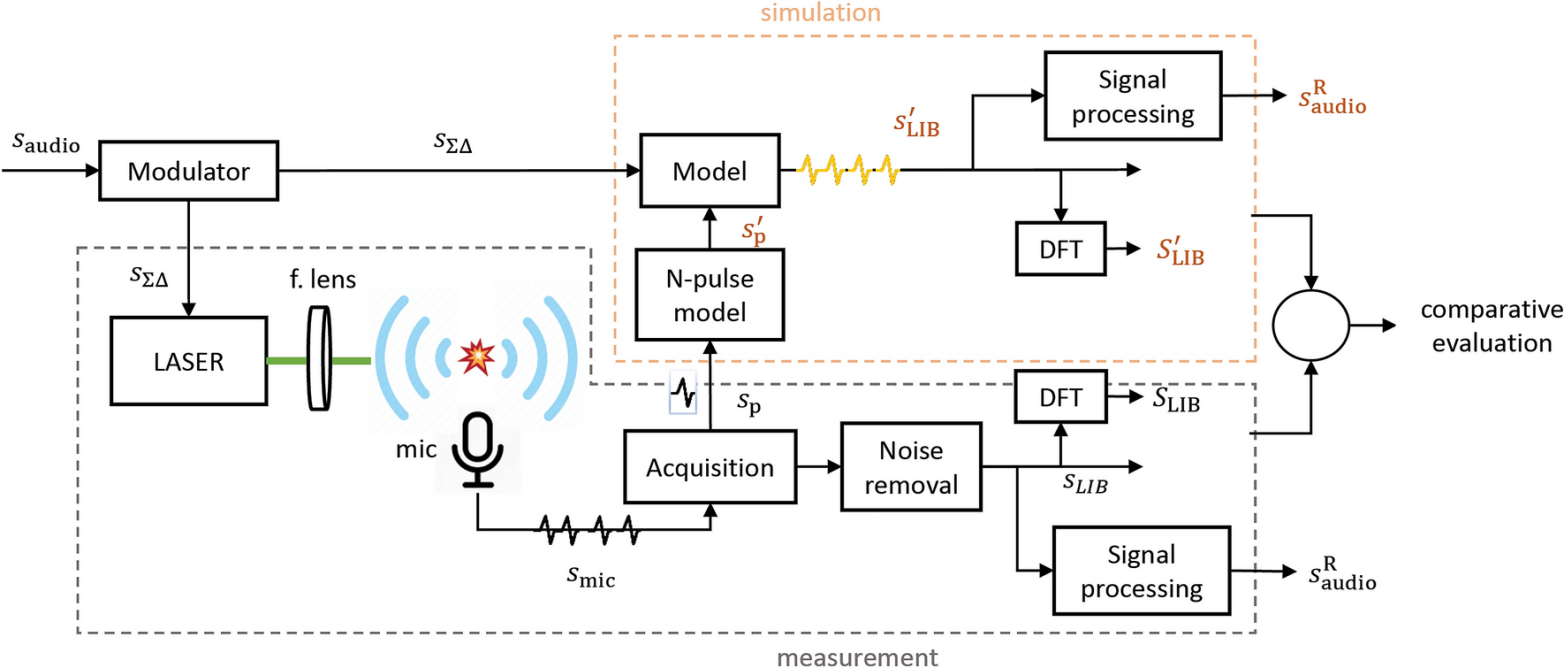
Methodology block diagram for the experimental and computational evaluation of the laser-audio transducer prototype.

For the evaluation of the proposed laser-sound prototype system, a parallel experimental and modeling procedure was adopted, which is outlined in Fig. 2. The input signal ${s}_{\mathrm{audio}}(n)$, with $n$ being the discrete-time index, is typically obtained from a typical Pulse Code Modulation (PCM) audio file and is routed to the modulator, where it is transformed into a ΣΔ bitstream. The modulator output ${s}_{{\Sigma \Delta } }\left(n\right)$ is used to control the laser emission and as input for the computational model. In the physical system, the optical pulses are focused in the air inducing breakdown and generating acoustic pulse trains, which are captured and recorded by a microphone and data acquisition system. The captured signals ${s}_{\mathrm{mic}}(n)$ are post-processed in order to reduce measurement artefacts and noise, resulting in the signal ${s}_{\mathrm{LIB}}\left(n\right)$. The frequency spectra ${S}_{\mathrm{LIB}}(k)$ of the reproduced acoustic pulse streams are obtained here by means of the Discrete Fourier Transform (DFT), where $k$ is the discrete frequency index. Moreover, single laser-generated acoustic pulses ${s}_{\mathrm{p}}(n)$ are acquired and analyzed to produce the signal model ${s}_{\mathrm{p}}^{{\prime}}(n)$ of the acoustic N-pulse. The signal ${s}_{\mathrm{p}}^{{\prime}}(n)$ is used as input for the computational model, which produces the simulated laser-generated acoustic signals ${s}_{\mathrm{LIB}}^{{\prime}}\left(n\right)$ and their respective DFT representation ${S}_{\mathrm{LIB}}^{{\prime}}(k)$. Finally, the signals ${s}_{\mathrm{LIB}}\left(n\right)$ and ${s}_{\mathrm{LIB}}^{{\prime}}\left(n\right)$ are low-pass filtered and resampled to the effective bandwidth of the system, leading to the reconstructed audio signals ${s}_{\mathrm{audio}}^{\mathrm{R}}\left(n\right)$. The signals ${s}_{\mathrm{audio}}^{\mathrm{R}}\left(n\right)$ are used for aural evaluations of the reproduced audio signals. It is important to note that, in the physical system, in analogy to the low-pass filter applied on the represented signals, the filtering of the high frequencies takes place during the sound propagation in the air^37^, as well as by the upper frequency limit of the human auditory system. Since the human auditory system does not perceive frequencies above 20 kHz, a physical laser-sound system with a band of interest extending up to 20 kHz would effectively reconstruct the audio signal without perceptible out-of-band distortions. An example of such a system is given in the Simulation of the ideal optoacoustic transducer subsection.

### Computational model

The proposed mathematical model of the laser-driven sound reproduction via ΣΔ audio streams allows for the evaluation of any reproduced signal ${s}_{\mathrm{LIB}}^{{\prime}}\left(n\right)$ from systems with arbitrary technical characteristics, i.e. laser repetition rates and optical pulse parameters. Therefore, the model is suitable for defining the laser specifications and the modulation scheme for the design of laser-sound systems. In the model, the reproduced signal is expressed in terms of the modulated signal ${s}_{{\Sigma \Delta }}(n)$ and the N-pulse signal ${s}_{\mathrm{p}}(n)$. For this reason, the time–frequency characteristics of the modulator and the N-pulse signals need to be analysed for the development of the model.

#### ΣΔ modulator

Assuming an oversampled Pulse-Code Modulation (PCM) input signal ${s}_{\mathrm{audio}}(n)$ with ${\rm N}$ number of samples, the discrete-time PCM-to-ΣΔ conversion can be described as a transformation ${\mathrm{M}}_{\mathrm{SD}}$ from ${s}_{\mathrm{audio}}(n)$ to the modulator output ${s}_{{\Sigma \Delta }}(n)$:

1 $${s}_{\Sigma \Delta }(n)={M}_{\Sigma \Delta }\left\{{s}_{audio}(n)\right\}$$

In the discrete frequency domain obtained by DFT, the output ${S}_{{\Sigma \Delta }}(k)$ of a ΣΔ modulator with respect to the input ${S}_{\mathrm{audio}}(k)$ can be generally written as:

2 $${S}_{\Sigma \Delta }\left(k\right)=STF\left(k\right)\cdot {S}_{audio}\left(k\right)+NTF\left(k\right)\cdot E\left(k\right)$$

where $k$ is the discrete frequency index, $\mathrm{STF}\left(k\right)$ and $\mathrm{NTF}\left(k\right)$ are the noise and signal transfer functions of the modulator^30^ respectively, and $E(k)$ is the quantization noise. Considering 1-bit ΣΔ, there are many different implementations depending on the order of the integrator loop^30^ of the modulator (see Methods section for details). Here, for demonstration purposes, a first-order ΣΔ modulator was adopted for which $\mathrm{STF}\left(k\right)=1$ and $\mathrm{NTF}\left(k\right)=1-{e}^{-j\frac{2\pi }{N}k}$, thus:

3 $${S}_{\Sigma \Delta }\left(k\right)={S}_{audio}\left(k\right)+(1-{e}^{-j\frac{2\pi k}{N}})\cdot E(k)$$

The spectral magnitude of the first order NTF can be written as:

4 $$\left|NTF\left(k\right)\right|=2\cdot \left|\mathit{sin}\left(\frac{\pi }{N}k\right)\right|$$

For the inband frequencies, $k\ll N$ and Eq. (4) can be approximately written as:

5 $$\left|NTF\left(k\ll N\right)\right|\simeq \frac{2\pi }{N}k$$

where $N$ is the frequency index of the sampling frequency. Equation (5) shows that the quantization noise of a first order ΣΔ modulator exhibits approximately a first order high-pass spectral profile in the inband. For this modulator and a full-scale sine wave input, the Signal-to-Quantization-Noise Ratio (SQNR) is:

6 $$SQNR=\frac{9{L}^{3}}{2{\pi }^{2}} (dB)$$

where $L$ is the oversampling ratio.

#### Laser-generated acoustic pulse model

Laser-generated acoustic waves are generally modeled as ideal N-pulses^18,22^. By adopting the approach presented in Kaleris et al.^23^, the modeled N-pulse signal ${s}_{\mathrm{p}}^{{\prime}}(n)$ is expressed as:

7 $${s}_{p}^{{\prime}}\left(n\right)=\frac{A}{{N}_{p}}\cdot n\cdot \left(u\left(n+{N}_{p}\right)-u\left(n-{N}_{p}\right)\right)$$

where $u(n)$ is the unit step function, ${N}_{p}$ is the number of half the samples of the pulse and $A$ is the pulse amplitude. The frequency spectrum ${S}_{\mathrm{p}}^{\prime}(k)$ of ${s}_{\mathrm{p}}^{{\prime}}\left(n\right)$ obtained via DFT can be written as:

8 $${S}_{p}^{{\prime}}\left(k\right)=\frac{{\rm A}}{{N}_{p}}\cdot \sum_{n=-{N}_{p}}^{{N}_{p}}n\cdot {e}^{-j\frac{2\pi }{N}kn}$$

Due to the very short duration of the N-pulse, which is in the order of a few tens of microseconds, ${N}_{p}$ takes very small values compared to $N$, so that the spectral magnitude $\left|{S}_{\mathrm{N}}^{{\prime}}\left(k\right)\right|$ of the laser-generated N-pulse becomes:

9 $$\left|{S}_{p}^{{\prime}}\left(k\right)\right|=A\cdot \left|\mathit{sin}\left(\frac{2\pi {N}_{p}}{N}k\right)\right|$$

Again, for the frequencies in the band of interest, $k\ll N$ and $\frac{{N}_{\mathrm{p}}}{N}k\ll 1$, Eq. (9) becomes:

10 $$\left|{S}_{p}^{{\prime}}\left(k\right)\right|\simeq \frac{2\pi A{N}_{p}}{N}k$$

Equation (10) shows that for the inband frequencies, the laser-generated acoustic pulses exhibit a first-order high-pass spectral profile. This characteristic profile holds for the major part of the parameter range of the generating laser pulses^18,22,24^ and, as presented in the next paragraph, it shapes the frequency response of the laser-audio system.

#### Simulation of laser-driven audio reproduction

To simulate the laser-generated acoustic pulse streams, the output of the ΣΔ modulator is represented in the discrete-time domain as a stream of impulses:

11 $${s}_{\Sigma \Delta }\left(n\right)=\sum_{i=1}^{{N}_{\delta }}\delta \left(n-{n}_{i}\right)$$

where $\delta \left(\cdot \right)$ is the Kronecker delta function, ${n}_{i}$ is the time index of the ${i}_{th}$ delta and ${N}_{\updelta }$ the total number of impulses in the modulated signal. Given that each ΣΔ impulse triggers a single acoustic N-pulse, the acoustic pulse stream is described by Eq. (11) via convolution of the modeled N-pulse ${s}_{\mathrm{p}}^{{\prime}}(n)$ with the modulator output ${s}_{{\Sigma \Delta }}\left(n\right)$:

12 $${s}_{LIB}^{{\prime}}\left(n\right)={s}_{\Sigma \Delta }\left(n\right)*{s}_{p}^{{\prime}}\left(n\right)=\sum_{i=1}^{{N}_{\delta }}\delta \left(n-{n}_{i}\right)*{s}_{p}^{{\prime}}\left(n\right)$$

By substituting Eq. (7) in (12), we get:

13 $${s}_{LIB}^{{\prime}}\left(n\right)={s}_{\Sigma \Delta }\left(n\right)*{s}_{p}^{{\prime}}\left(n\right)=\frac{A}{{N}_{p}}\cdot \left(\sum_{i=1}^{{N}_{\delta }}\delta \left(n-{n}_{i}\right)\right)*\left(n\cdot \left(u\left(n+{N}_{P}\right)-u\left(n-{N}_{P}\right)\right)\right)$$

The DFT spectrum ${S}_{\mathrm{LIB}}^{{\prime}}\left(k\right)$ of the laser-generated pulse stream is the result of the multiplication of the frequency spectra ${S}_{{\Sigma \Delta }}\left(k\right)$ of the ΣΔ bitstream by ${S}_{\mathrm{p}}^{{\prime}}\left(k\right)$:

14 $${S}_{LIB}^{{\prime}}\left(k\right)={S}_{\Sigma \Delta }\left(k\right)\cdot {S}_{p}^{{\prime}}\left(k\right)={S}_{p}^{{\prime}}\left(k\right)\cdot {S}_{audio}\left(k\right)+{S}_{p}^{{\prime}}\left(k\right)\cdot NTF\left(k\right)\cdot E\left(k\right)$$

Since ${S}_{\mathrm{p}}^{{\prime}}\left(k\right)$ exhibits a first-order high-pass profile in the inband frequency range (see Eq. (10)), the spectral magnitude of the signal transfer function for the laser-audio system becomes:

15 $$\left|{STF}_{LIB}\left(k\right)\right|=\left|{S}_{p}^{{\prime}}\left(k\right)\right| \sim k$$

while the resulting noise transfer function becomes:

16 $$\left|{NTF}_{LIB}\left(k\right)\right|=\left|{S}_{p}^{{\prime}}\left(k\right) \right|\cdot \left| NTF\left(k\right)\right| \sim {k}^{2}$$

which corresponds to a second-order high-pass profile. As shown in the next subsection, Eqs. (14)–(16) are clearly demonstrated by the simulated and measured signals.

### Experimental evaluation of the prototype system

In the experimental setup, the parameters and characteristics of the 1-bit ΣΔ modulation were largely dictated by the specifications of the laser system that was available for the implementation of the transducer prototype. The laser system allowed for a maximum optical pulse repetition rate of 20 kHz, however, a repetition rate ${f}_{\mathrm{laser}}=4\,\mathrm{kHz}$ was adopted for the experiments where the targeted material was the air. This because for rates higher than 4 kHz, the optical energy dropped close to the breakdown threshold and some optical pulses did not cause air breakdown, leading to “missing pulses”. For such low repetition rates, the pulse-to-pulse time distance (${t}_{ptp}\ge$ 250 μs) is sufficiently large for the plasma to relax and the interaction volume to cool down. The plasma lifetime, as well as the duration of the thermoelastic expansion and collapse depend on the laser radiation parameters, such as pulse duration, wavelength, energy and focusing conditions^10,11^. For laser parameters identical to those used in this prototype system, it has been observed that the plasma relaxes within a few tens of nanoseconds, while the full thermoelastic process relaxes within a few tens of microseconds^22^. As a result, each laser pulse of the ΣΔ pulse train is focused in neutral air of identical temperature and density, a fact that manifests also in the high repeatability of the acoustic pulses.

Due to the restriction in the available repetition rate, and in order to effectively demonstrate the functionality of the system at a proof-of-concept level, a ΣΔ oversampling factor of $L=2$ was adopted, together with a first-order noise shaping. Consequently, the original sampling frequency of the input signals was set to ${f}_{\mathrm{s}}=2\, \mathrm{kHz}$ (${f}_{\mathrm{laser}}/L$) allowing for a useful signal frequency range up to 1 kHz according to the Nyquist criterion: ${f}_{\mathrm{audio}}\in [20\, \mathrm{Hz}, 1 \,\mathrm{kHz}]$. Although such restrictions compromise the frequency range of the signals, the results clearly illustrate the principles introduced by this work and are adequate for validating the computational model. For this purpose, the experimental and computational results for a single sine wave input are presented and compared in the next subsections. Moreover, the computational model is used to simulate the reproduction of a sine wave signal by an ideal optoacoustic transducer designed according to the specifications of high-performace commercially available laser systems, which allow for significantly higher repetition rates. The simulated acoustic pulse train is reconstructed in the time domain via signal processing, to demonstrate the possibility of direct demodulation in the air. Finally, it is noted that, apart from the results presented in the next paragraphs, tests with typical sine sweep, speech and music signals have verified the expected audio reproduction capabilities of the proposed system. Recorded samples of such signals can be accessed at the supplementary material of this work.

#### Single sine wave reproduction

**Figure 3:**
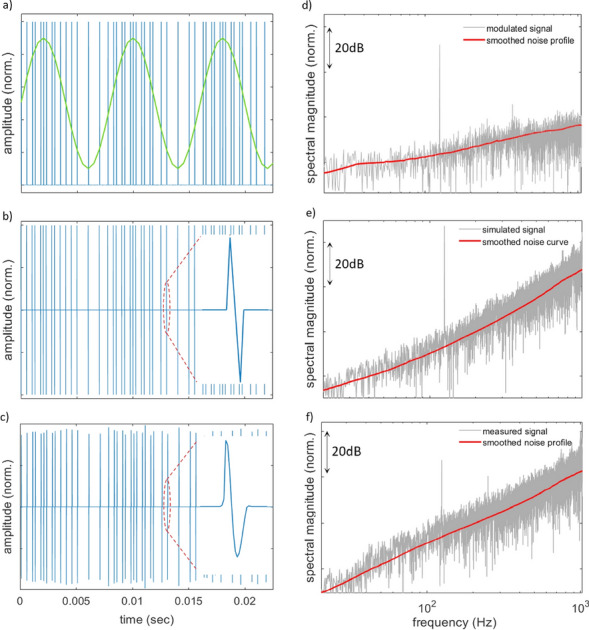
Time-domain representations of a 125 Hz sine wave signal (**a**) encoded as a ΣΔ stream ${s}_{{\Sigma \Delta }}(n)$ of rectangular pulses, (**b**) simulated as a LIB pulse stream ${s}_{\mathrm{LIB}}^{{\prime}}(n)$ using the modeled ideal N-pulse signal ${s}_{\mathrm{p}}^{{\prime}}(n)$ and (**c**) reproduced from the laser-audio system ${s}_{\mathrm{LIB}}(n)$, (**d**–**e**) frequency-domain representations of the signals within the inband range $[20\,\mathrm{Hz}, 1\,\mathrm{kHz}]$, as obtained via DFT.

Sine wave signals are commonly used as stimuli for the evaluation of acoustic systems and transducers^38^. Since sine waves concentrate all of their energy in one frequency, they are also suitable for evaluating digital modulations, especially for low SQNR conditions, as is the case for the presented prototype transducer. Figure 3a shows a part of a sinusoidal signal with frequency ${f}_{\mathrm{sine}}=125\,\mathrm{Hz}$, superimposed on its ΣΔ representation that is produced after the modulator stage. This ideal ΣΔ modulated sine wave is compared with the respective signal ${s}_{\mathrm{LI}\mathrm{B}}^{{\prime}}(n)$ as evaluated by the computational model (Fig. 3b) and the measured signal ${s}_{\mathrm{LI}{\rm B}}(n)$ that is generated by the prototype system (Fig. 3c). By observation of the Figs. 3a–c, it can be seen that the modulated, simulated and measured signals all represent the same pulse train. It becomes apparent that the laser-audio system is able to reproduce the ΣΔ pulse sequences with high accuracy, essentially replacing the rectangular ΣΔ pulses with laser-generated N-pulses. Accordingly, it is also shown that the computational model accurately represents the measured signal.

Figures 3d–f show the corresponding frequency spectra of the signals as obtained via DFT within the inband frequency range. In the spectrum ${S}_{{\Sigma \Delta }}(k)$ of the ideal ΣΔ signal (Fig. 3d), the sine wave frequency is prominent at 125 Hz, approximately 35 dB higher than the noise floor in the neighboring frequencies while the characteristic noise shaping of the modulator can be observed as a first-order high-pass slope of the quantization noise floor. Figure 3e shows the spectrum ${S}_{\mathrm{LIB}}^{{\prime}}(k)$ of the simulated signal ${s}_{LIB}^{{\prime}}(n)$, as obtained by multiplying ${S}_{{\Sigma \Delta }}(k)$ by the spectrum ${S}_{\mathrm{p}}^{{\prime}}(k)$ of a single modeled N-pulse. From Fig. 3e it can be seen that the spectral magnitude of the sine wave frequency is preserved, while the noise floor of ${S}_{\mathrm{LIB}}^{{\prime}}(k)$ has a second-order high-pass profile as a result of the combined effect of the ΣΔ noise shaping and the spectrum ${S}_{\mathrm{p}}^{{\prime}}(k)$ of the N-pulse (see Eq. (14)). The same spectral profile also manifests in the spectrum ${S}_{\mathrm{LIB}}(k)$ of the measured signal shown in Fig. 3f, but here the noise floor is raised by approximately 10 dB, due to the acoustic noise introduced in the experiment. Also, in ${S}_{\mathrm{LIB}}(k)$ the second harmonic of the sine wave frequency can be seen in ${S}_{\mathrm{LIB}}(k)$, which indicates a potential mild non-linearity in the reproduction chain.

Nevertheless, laser-sound systems are capable of generating multi-level pulse streams with increased quantization resolution, which effectively leads to an improved SQNR. This approach requires real-time modulation of the laser pulse energy and an excess of optical power to achieve optical breakdown well-below the maximum output of the laser. Although not available for the present work, such laser systems are commercially available and become increasingly accessible with time. In the next subsection, a multi-level laser-sound system is simulated with the use of the mathematical model, demonstrating a full-bandwidth, high-fidelity optoacoustic transducer.

#### Simulation of the ideal optoacoustic transducer

In the previous subsection, it was demonstrated that the computational model provides accurate predictions of the measured signal—albeit with the discussed limitations compared to the ideal requirements. Hence, it is now feasible to use the model to simulate the performance of an ideal optoacoustic transducer that is capable of reproducing continuous sound over the entire audible frequency range with high fidelity. To demonstrate the functionality of such an ideal optoacoustic transducer, we consider a state-of-the-art commercially available laser unit with ${f}_{\mathrm{laser}}=160\,\mathrm{kHz}$ pulse repetition rate and sufficient pulse intensity to induce breakdown in air, see for example^39,40^. Given the audible frequency range ${f}_{\mathrm{audio}}\in [20\,\mathrm{ Hz}, 20\,\mathrm{ kHz}]$, the initial sampling frequency of the input signal is now selected to be ${f}_{s}=40 \,\mathrm{kHz}$. Under these conditions, the repetition rate of the laser allows for an oversampling factor $L=\frac{{f}_{\mathrm{laser}}}{{f}_{\mathrm{s}}}=4$, while a 5-bit, 6th-order ΣΔ modulator with pole placement optimization is deployed used to further reduce SQNR in the band of interest^30^. It should be noted that, in a real system, the multi-level functionality can be achieved with high precision by using an electro-optic modulator^41^. To account for the multi-level ΣΔ stream, Eq. (11) of the computational model has to be adapted as:

17 $${s}_{\Sigma \Delta }\left(n\right)=\sum_{i=1}^{{N}_{\delta }}{a}_{i}\cdot \delta \left(n-{n}_{i}\right)$$

where ${a}_{i}\in [\mathrm{0,1}]$ the quantized amplitude of the ${i}_{th}$ delta function.

**Figure 4:**
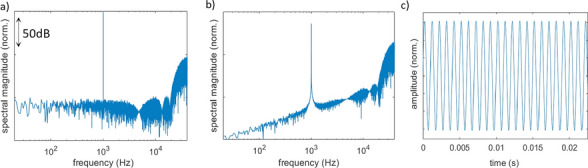
(**a**) ${S}_{{\Sigma \Delta }}\left(n\right)$, (**b**) ${S}_{\mathrm{LIB}}^{{\prime}}\left(n\right)$ and (**c**) ${s}_{\mathrm{audio}}^{\mathrm{R}}\left(n\right)$ for a 1 kHz sine wave input, simulated under consideration of an ideal optoacoustic transducer based on a state-of-the-art 160 kHz pulsed laser system.

Figure 4 shows the simulation results of the reproduction of a 1 kHz sine wave from the ideal laser-sound system. Figure 4a shows the DFT spectrum ${S}_{{\Sigma \Delta }}(f)$ of the modulator output, where the optimized pole placement can be seen at the high frequencies of the audible spectrum, while the noise is shaped above 20 kHz. Due to the pole optimization of the modulator, the quantization noise floor becomes flat in the inband range^30^: $\mathrm{NTF}\left(k\right)=1, k\in [0,N]$. Figure 4b shows the simulated spectrum ${S}_{\mathrm{LIB}}^{{\prime}}(k)$ of the laser-generated acoustic pulse stream, where the NTF takes the predicted first-order high-pass profile as a result of multiplication by the spectral profile of the N-pulse (see Eqs. (14)–(16)):

$${\mathrm{NTF}}_{\mathrm{LIB}}\left(k\right)\sim k$$

Figure 4c shows the reconstructed time-domain signal ${s}_{\mathrm{audio}}^{R}(n)$ which is produced by filtering of the simulated signal ${s}_{\mathrm{LIB}}^{{\prime}}\left(n\right)$ with a low-pass filter that has a cutoff frequency at ${f}_{\mathrm{filt}}=20\,\mathrm{kHz}$ (see also section Methods). The reconstructed signal has the time profile of a perfect sine wave, demonstrating the fact that the laser-generated pulse train maintains the information of the input signal in the band of interest and can be recovered by simple low-pass filtering.

It has to be noted that the high pulse repetition rate adopted in this simulation could potentially impose difficulties in a real implementation of a system with equivalent specifications. Here, the minimum pulse-to-pulse time distance is ${t}_{ptp}=6.25$ μs, while the relaxation time of the thermoelastic phenomenon^21,22^ is in the order of several tens of microseconds. Considering two consecutive laser pulses, the conditions of the air in the focal spot at the moment of arrival of the second pulse, such as the temperature and pressure and particle density, will deviate from equilibrium^10,11^. For even higher laser repetition rates, in the order of several MHz, the second pulse will be focused in air plasma, leading to different focusing conditions and light-matter interaction phenomena. Double pulse air breakdown has been studied in several works^10,11,42^, from which it becomes evident that the optical absorption of the second pulse is significantly enhanced, leading to a stronger thermoelastic phenomenon. As a result, the acoustic behavior of a plasma sound source with such a fast, consecutive excitation will deviate from the behavior described here. However, due to the complexity of the phenomenon, in-depth analysis is necessary for the description of the generated sound. In order to avoid this unexpected behavior, several solutions could be adopted, such as:

- the use of ultra-short laser pulses to reduce the duration of the thermoelastic phenomenon,
- rapid pulse-to-pulse micro-shifting of the laser focus to direct each pulse at different positions in the air,
- the use of specially designed solid targets, such as rotating metal disks.

Nevertheless, double- or multi-pulse radiation schemes could also be considered for the shaping of complex sound waves, as well as for the increase of the total efficiency of the optoacoustic transduction.

## Discussion

A novel laser-based optoacoustic transducer was presented that is capable of controlled and predictable generation of acoustic signals. The reproduction was based on the encoding of audio information into 1-bit or multibit ΣΔ pulse streams, which were materialized directly on the target medium as trains of laser-generated acoustic pulses via laser-induced breakdown. To the best of the authors’ knowledge, this is the first report of a working prototype and a complete mathematical model describing laser-driven reproduction of arbitrarily complex continuous sound waves without the need for a demodulation device. The functionality of the transducer was demonstrated at a proof-of-concept level (Technical Readiness Level 3) by experimental evaluation of the reproduction of sinusoidal signals. The experimental results were supported by computational evaluations estimating the reproduced audio signal as the result of the convolution of the driving ΣΔ bitstream with the signal profile of a single laser-generated acoustic pulse. Nonetheless, the system is capable of reproducing acoustic signals of arbitrary complexity, since ΣΔ encoded signals are only restricted by the modulator’s bandwidth and not by the signal’s form. Also, the laser can respond to any triggering sequence encoded into a ΣΔ bitstream within the limits of its maximum pulse repetition rate, which defines the effective bandwidth of the system. Thus, the results presented for the special case of single sine waves can be generalized to arbitrarily complex signals within the bandwidth of the system. In order to demonstrate this aspect, recorded samples from the reproduction of complex speech and music signals are provided as supplementary material. The evaluations were carried out within a limited frequency range of the audible spectrum due to technical limitations of the available experimental resources, however, the extension of the reproduction frequencies to the full audible spectrum or the ultrasounds emerges directly from the presented results by increasing the laser pulse repetition rate. Such an extension was investigated by means of the computational model that was used to simulate an ideal optoacoustic transducer considering a laser unit with state-of-the-art specifications.

Laser-sound opens up new horizons for audio reproduction as it enables the unbounded positioning of massless sound sources within the listening space, by focusing the laser beam in the air or on specially coated solid surfaces. The digital ΣΔ modulated bitstreams triggering the laser are directly demodulated into acoustic waves without the need for digital-to-analog conversion, wired connections and power-consuming electromechanical transduction units. With the use of moving focus techniques, such as moving mirrors, fast shifting of the acoustic source can be achieved for real-time rendering of moving sound objects. The proposed technology is also suitable for remote sound reproduction via the transmission and direct demodulation of signals over very long distances, without the need for local power supply, as the optical pulse stream carries both the audio information and the power required for the reproduction. Fast generation of plasma sound sources inside narrow-band acoustic resonators placed at arbitrary distances from the laser source can also be adopted to appropriately filter the generated signal. Moreover, the support of direct sound reproduction from digital modulations allows for the implementation of fully digital audio reproduction chains without moving parts and with potential directivity control via virtual volumetric arrays^26^. In contrast to the electromechanical transducers, the in band frequency response of optoacoustic transducers is well-defined and stable, as the system is free of moving parts and is not subject to constructional or material defects. Moreover, the plasma sources are capable of generating strong broadband impulse-like signals which are useful in applications where the rendering of rapid sound events is required, as for example in acoustic measurements. Finally, it is anticipated that laser-sound technology can reach or even surpass the current electromechanical technology in terms of power efficiency, which is less than 2% for the direct emission of the typical moving-coil commercial devices, while the theoretical maximum is below 4%^43^. It is estimated that an optimized laser-sound system can achieve an efficiency higher than $4\%$, depending on the wall-plug efficiency of the driving laser unit, the optical absorption efficiency and the optical-to-sound coupling. The latter is intrinsic to the optoacoustic transduction and depends on the parameters of the laser pulse, however, there are only scarce mentions to the particular dependence in the bibliography^18^ and a systematic study should be carried out in the future.

Based on these advantages, the long-term vision of this work is the reproduction of controlled holographic sound via laser-driven spatially unbounded virtual sound sources at varying distances from the optical source within the limits imposed by the required focusing conditions, without the need for localized transduction devices. The envisaged laser-sound system will be able to precisely reconstruct any desired sound projection pattern, with sufficient power and well-defined time–frequency acoustic characteristics that make it suitable for future all-digital holographic sound reproduction systems. The modulated laser data streams could transmit both useful communication signals and the power required for distant sound reproduction. For the adoption of the technology in commercial applications, there are three main obstacles that have to be overcome. Currently, state-of-the-art lasers capable of generating breakdown at high repetition rates are costly, however, their price is gradually decreasing due to progress in laser technology and a quick increase in their demand for use in scientific and commercial applications. Also, safety issues due to direct, reflected or scattered optical radiation from the laser have to be addressed in installations where there is a possibility of skin or eye exposure. Finally, as presented in the Simulation of the ideal optoacoustic transducer subsection, for high laser repetition rates, the effect of consecutive excitation of the interaction volume before complete relaxation of the thermoelastic phenomenon could lead to acoustic behavior that deviates from the behavior outlined in the presented experiments and model. Nevertheless, due to its unique and unprecedented capabilities, laser-driven audio reproduction could become a complementary technology, or even an attractive alternative, to the established electromechanical transduction for a variety of commercial applications.

## Methods

### Experiments

**Figure 5:**
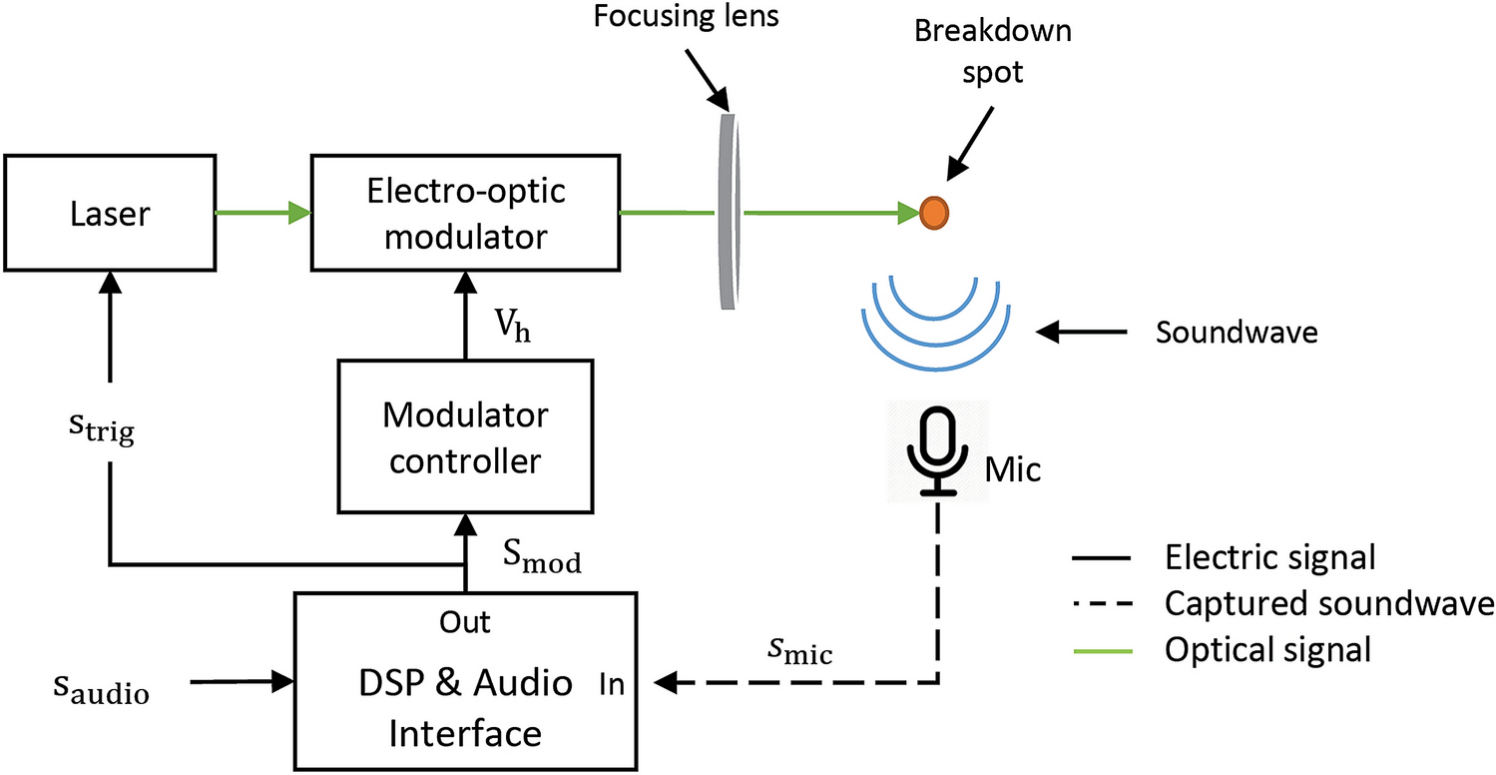
Schematic diagram of the optoacoustic transducer prototype.

The experimental procedure for setting, calibrating and evaluating the optoacoustic transducer prototype, is shown in Fig. 5. The core of the optoacoustic transducer constituted an Nd:YAG (EdgeWave IS-200-2-L) laser capable of emitting $532\, \mathrm{nm}$ pulses of $\sim 9\, \mathrm{mJ}$ energy and $\sim 10\, \mathrm{ns}$ duration at a repetition rate of $4 \,\mathrm{kHz}$. The pulse emission of the laser was triggered by the signal ${s}_{\mathrm{trig}}(t)$, implementing the ΣΔ optical pulse stream. The laser pulses were directed into an electro-optic modulator with the capability of real-time pulse-to-pulse control of the transmitted optical energy. The functionality of the modulator was controlled by a high-voltage source through the signal ${V}_{\mathrm{h}}(t)$. After the modulation, the pulses were focused in the air by 7.5 cm lens. A special microphone (B&K 4192) with high dynamic range of 19–162 dB and frequency response extending from the low infrasounds to the high audible frequencies $f\in \left[3\, \mathrm{Hz}, 20\, \mathrm{kHz}\right]$ was placed at a distance of 5 cm from the breakdown spot. The microphone signal was routed into a sound card with broad frequency response and high sampling rate (RME Adi-2 Pro) at ${f}_{\mathrm{s}}=384\, \mathrm{kHz}$ and 24-bit quantization resolution. The digital signal was recorded using the Audacity audio software^44^. Finally, it should be noted that the sound pressure level achieved via LIB depends on the laser pulse energy and can vary from very low, i.e. 45 dB, up to extremely high (130 dB or more). Therefore, it was necessary to use earplugs when experimenting with high-energy laser pulses.

### ΣΔ modulator structure

**Figure 6:**
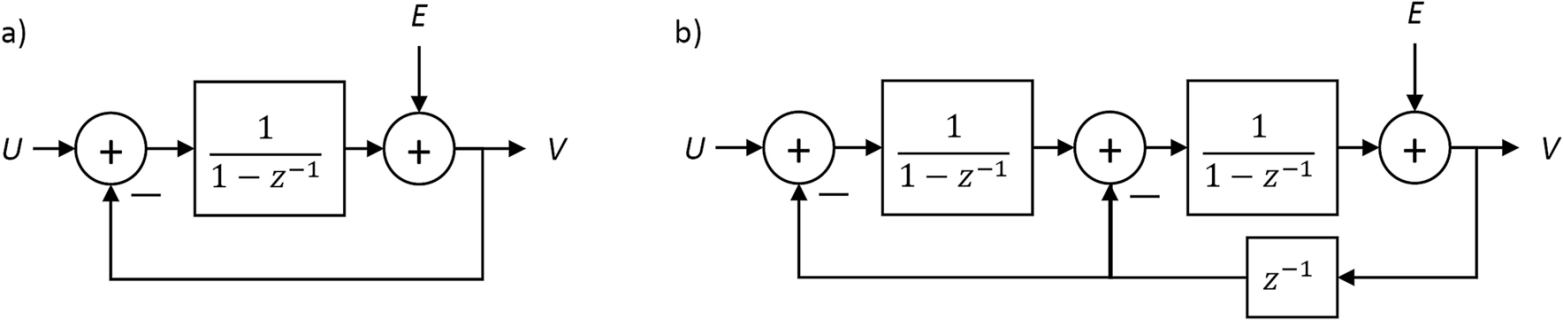
z-domain block diagrams of the linearized models of (**a**) a first-order and (**b**) a second order ΣΔ modulator, where $U, V$ the input and output signals respectively, $E$ is the quantization error and $z={e}^{-j\frac{2\pi }{N}k}$.

ΣΔ modulation entails negative feedback loops that involve integration and consequent quantization of the input signal. Quantization is carried out with low-resolution quantizers, usually 1 to 5 bits, leading to high quantization noise levels. This is treated with oversampling and noise shaping, through which a significant part of the noise energy is shifted out of the in band frequency range. The number of feedback loops used in a particular ΣΔ implementation, known as the order of the modulator, determines the shape of the noise transfer function and, consequently, the SQNR in the in band range. Figure 6 presents the block diagrams of the linearized models of a first-order and a second-order ΣΔ modulator in the discrete-frequency domain^30^. For the first-order modulator, the magnitude of the noise transfer function takes the form $\left|{\mathrm{NTF}}^{\left(1\right)}\left(k\right)\right|=2\left|\mathrm{sin}\left(\frac{\pi }{N}k\right)\right|$ while for the second-order modulator $\left|{\mathrm{NTF}}^{\left(2\right)}\left(k\right)\right|={\left(2\mathrm{sin}\left(\frac{\pi }{N}k\right)\right)}^{2}$. For $\frac{k}{N}\ll 2\pi$ corresponding to the in band frequencies, the NTFs reduce to $\left|{\mathrm{NTF}}^{\left(1\right)}\left(k\right)\right|\approx \frac{2\pi }{N}k$ and $\left|{\mathrm{NTF}}^{\left(2\right)}\left(k\right)\right|\approx {\left(\frac{2\pi }{N}k\right)}^{2}$. It becomes obvious that $\left|{\mathrm{NTF}}^{\left(2\right)}\left(k\right)\right|<\left|{\mathrm{NTF}}^{\left(1\right)}\left(k\right)\right|$ and thus, the second-order modulator achieves higher suppression of the quantization noise in the in band range. This result can be generalized for nth-order modulators; however, higher-order modulators^30^ suffer from stability issues which have to be addressed.

### Signal processing

For the acoustic evaluation of the system by means of measured and simulated audio signals, which are included as supplementary material, low-pass filtering was used to limit the frequency spectrum of the signals in the band-of-interest. In particular, the acoustic pulse trains ${s}_{\mathrm{LIB}}\left(n\right)$ and ${s}_{\mathrm{LIB}}^{{\prime}}\left(n\right)$ were filtered by an anti-aliasing low-pass filter ${s}_{\mathrm{filt}}(n)$, with cut-off frequency ${f}_{\mathrm{filt}}=\frac{{f}_{\mathrm{s}}}{2}$ equal to the Nyquist frequency of the original input signal. The band-limited signal was downsampled by a factor $M$ to a resulting frequency ${f}_{\mathrm{s}}$, to produce the reconstructed audio signal:

18 $${s}_{\mathrm{audio}}^{\mathrm{R}}\left(n\right)=\left({s}_{\mathrm{LIB}}*{s}_{\mathrm{filt}}\right)\left(nM\right)$$

**Figure 7:**
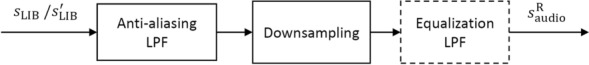
Signal processing steps for the production of the reconstructed signals.

Additionally, the complex audio signals, namely speech and music, were low-pass filtered throughout the inband range to equalize the high-pass profile of the system’s response (see Eq. (15)). In a full-specifications high-SQNR laser-sound system, the equalization can take place by preprocessing the input signal ${s}_{audio}$, however, further analysis of this aspect is beyond the scope of this work. Finally, the signal processing steps taken for the audio samples of the supplementary material are shown in Fig. 7. In the experiments, the following test signals were used:

1. single sine waves at the central frequencies of the octave bands^38^
2. band limited sine sweep signals
3. excerpt from female speech^45^ and music

Due to space restrictions, results from the signals of points 2 and 3 are not presented here. The resulting audio files are included as supplementary material and can be used for listening tests (Supplementary material audio_listening_tests.rar). For a detailed description of the audio files, see Supplementary material signals_details.pdf.

### Estimation of the transduction efficiency

Here, a step-by-step analysis of the total transduction efficiency of the prototype system is presented, along with a preliminary estimation of the optimal efficiency achieved by state-of-the-art components and optimized parameters. There are 3 sources of energy loss throughout the transduction chain: (a) electrical-to-optical conversion of the laser device, (b) optical energy absorption from the targeted medium and (c) optical-to-acoustic (thermal) energy conversion.

(a) The power consumption of the laser unit for a maximum optical output of 90 W at 532 nm wavelength is 1.2 kW, corresponding to an efficiency ${\eta }_{1}=7.5\%$. The efficiency doubles for infrared pulses (1064 nm).

(b) The efficiency of the optical energy absorption from the air depends on multiple parameters, particularly the pulse duration, energy, wavelength focusing conditions and air humidity and density. From measurements of the remaining optical energy after the focal spot it was found that in the presented experiments, about one third of the incoming optical energy is absorbed, thus ${\eta }_{2}=33\%$. However, due to the limitations of the available equipment, the optical intensity used here was close to the breakdown threshold, where the absorption efficiency is significantly reduced. In studies with higher optical intensities, absorption efficiencies of more than 80% have been observed. High absorption efficiencies can be also achieved by focusing the pulses on solid targets; however, such an analysis is beyond the scope of this work.

(c) Moreover, only part of the absorbed optical energy is converted into acoustical energy. This is due to losses in the form of optical and thermal radiation from the ionized volume, among others, that do not contribute to the thermoelastic expansion of the volume generating the pressure wave. The efficiency of this process can be estimated by calculating the total acoustic energy of the generated soundwave from the measured pressure, according to the formula:

$${E}_{\mathrm{s}}=\frac{4\pi {r}^{2}}{{\rho }_{0}c}\cdot \underset{0}{\overset{{T}_{\mathrm{p}}}{\int }}{p}_{\mathrm{N}}^{2}(t)dt$$

where ${E}_{\mathrm{s}}$ is the total acoustic energy, ${\rho }_{0}$ is the air density, $c$ is the speed of sound in air, $r$ is the measuring distance and ${p}_{\mathrm{N}}\left(t\right)$ is the sound pressure of the N-pulse and ${T}_{\mathrm{p}}$ the duration of the pulse. Using typical values for the particular laser parameters of the experiment, ${T}_{\mathrm{p}}\approx 60 \,\upmu \mathrm{s}$, peak pressure ${p}_{\mathrm{max}}=25\,\mathrm{Pa}$ at 1 m distance, results to 0.35 mJ total acoustic energy per pulse. For an 8 mJ pulse and 30% absorption, the absorbed energy is ${E}_{\mathrm{a}}=2.4\, \mathrm{mJ}$, which leads to:

$${\eta }_{3}=\frac{{E}_{\mathrm{s}}}{{E}_{\mathrm{a}}}=15\%$$

This result is in agreement with estimations given in [Oksanen]. According to the above, the total conversion efficiency of the experimental prototype is:

$$\eta ={\eta }_{1}{\eta }_{2}{\eta }_{3}=0.37\%$$

Assuming improved parameters and state-of-the-art devices ${\eta }_{1}=30\%$^46^$,{\eta }_{2}=80\%, {\eta }_{3}=20\%$, the efficiency would become:

$${\eta }_{\mathrm{imp}}=4.8\%$$

Note that the theoretical limit of the efficiency of moving-coil electromechanical transducers is ${\eta }_{\mathrm{em}}=4\%$ while the efficiency of common commercial transducers is ${\eta }_{\mathrm{com}}=2\%$^43^.

## Supplementary Information

Below is the link to the electronic supplementary material.

Supplementary Material 1.

Supplementary Material 2.

## References

[1] Beranek, L. L. & Mellow, T. J. *Acoustics: Sound Fields, Transducers and Vibration* (Academic Press, London, 2019).

[2] Sanders, R. R. *The Electrostatic Loudspeaker Design Cookbook* (Audio Amateur Pr, Peterborough, NH, 2004).

[3] The Piezo-Electric Loud-Speaker. *Nature* **133**, 184 (1934).

[4] Tian, Y. *et al.* Coherent generation of photo-thermo-acoustic wave from graphene sheets. *Nat. Sci. Rep.* **5**, 10582 (2015). 10.1038/srep10582 PMC4650607 26053560

[5] Kontomichos, F., Koutsioubas, A., Mourjopoulos, J., Spiliopoulos, N. & Vradis, A. A thermoacoustic device for sound reproduction. *J. Acoust. Soc. Am.* **123**(5), 3707 (2008).

[6] Béquin, P., Castor, K., Herzog, P. & Montembault, V. Modeling plasma loudspeakers. *J. Acoust. Soc. Am.* **121**(4), 1960–1970 (2007). 17471712 10.1121/1.2697201

[7] Shah, S. K. H. *et al.* Laser induced breakdown spectroscopy methods and applications: a comprehensive review. *Radiat. Phys. Chem.* **170**, 108666 (2020).

[8] Killiny, N. *et al.* Laser-induced breakdown spectroscopy (LIBS) as a novel technique for detecting bacterial infection in insects. *Sci. Rep.* **9**, 2449 (2019). 30792483 10.1038/s41598-019-39164-8 PMC6385218

[9] Welch, D. R. & Miley, G. H. Shock wave behavior in inertial confinement fusion implosions. In *Laser Interaction and Related Plasma Phenomena* (eds Hora, H. & Miley, G. H.) (Springer, Boston, MA, 1986).

[10] Papeer, J. *et al.* Multi variable control of filamentation of femtosecond laser pulses propagating in air. *J. Phys. B At. Mol. Opt. Phys.* **48**, 094005 (2015).

[11] Henis, Z., Milikh, G., Papadopoulos, K. & Zigler, A. Generation of controlled radiation sources in the atmosphere using a dual femtosecond/nanosecond laser pulse. *J. Appl. Phys.* **103**, 103111 (2008).

[12] Jones, T., Hornstein, M., Ting, A. & Wilkes, Z. Intense underwater laser acoustic source for Navy applications. *J. Acoust. Soc. Am.* **125**, 2556–2556 (2009).

[13] Hornstein, M., Jones, T., Ting, A. & Nicholas, M. Directivity and frequency control of an intense underwater laser acoustic source for navy applications. *J. Acoust. Soc. Am.* **127**, 1985–1985 (2010).

[14] Nelson, P., Veyrie, P., Berry, M. & Durand, Y. Experimental and theoretical studies of air breakdown by intense pulse of light. *Phys. Lett.* **13**, 226–228 (1964).

[15] Zel’dovič, J. & Rajzer, J. *Physics of Shock Waves and High-Temperature Hydrodynamic Phenomena* (Academic Press, London, 1970).

[16] Chen, J., Ni, X., Lu, J. & Bian, B. Initial formation process of laser-induced plasma shock wave in air. *Opt. Commun.* **176**(4–6), 437–440 (2000).

[17] Zhang, Z. & Gogos, G. Theory of shock wave propagation during laser ablation. *Phys. Rev. B* **69**, 235403 (2004).

[18] Oksanen, M. & Hietanen, J. Photoacoustic breakdown sound source in air. *Ultrasonics* **32**, 327–331 (1994).

[19] Ghosh, S. & Mahesh, K. Numerical simulation of laser-induced breakdown in air, 46th AIAA Aerospace Sciences Meeting and Exhibit (2008).

[20] Qin, Q. & Attenborough, K. Characteristics and application of laser-generated acoustic shock waves in air. *Appl. Acoust.* **65**(4), 325–340 (2004).

[21] Harilal, S., Brumfield, B. & Phillips, M. Lifecycle of laser-produced air sparks. *Phys. Plasmas* **22**, 063301 (2015).

[22] Kaleris, K., Orphanos, Y., Bakarezos, M., Dimitriou, V., M., Tatrakis, M., Mourjopoulos, J. & Papadogiannis, N. On the correlation of light and sound radiation following laser-induced breakdown in air. *J. Phys. D: Appl. Phys.* **53**, 435207 (2020).

[23] Kaleris, K., Orfanos, Y., Bakarezos, M., Papadogiannis, N. & Mourjopoulos, J. Experimental and analytical evaluation of the acoustic radiation of femtosecond laser plasma filament sound sources in air. *J. Acoust. Soc. Am.* **146**, EL212–EL218 (2019). 31590509 10.1121/1.5124509

[24] Gómez Bolaños, J., Delikaris-Manias, S., Pulkki, V., Eskelinen, J. & Hæggström, E. Laser-induced acoustic point source for accurate impulse response measurements within the audible bandwidth. *J. Acoust. Soc. Am.* **135**, EL298–EL303 (2014). 24907837 10.1121/1.4879664

[25] Gómez-Bolaños, J. *et al.* Benefits and applications of laser-induced sparks in real scale model measurements. *J. Acoust. Soc. Am.* **138**, EL175–EL180 (2015). 26428809 10.1121/1.4929623

[26] Eskelinen, J., Hæggström, E., Delikaris-Manias, S., Bolaños, J. & Pulkki, V. Beamforming with a volumetric array of massless laser spark sources—application in reflection tracking. *J. Acoust. Soc. Am.* **137**, 389–395 (2015). 10.1121/1.4920970 26093445

[27] Bennett, W. R. New results in the calculation of modulation products. *Bell Syst. Tech. J.* **12**(2), 228–243 (1933).

[28] Song, Z. & Sarwate, D. V. The frequency spectrum of pulse width modulated signals. *Signal Process.* **83**(10), 2227–2258 (2003).

[29] Floros, A. & Mourjopoulos, J. Distortion-free 1-bit PWM coding for digital audio signals. *EURASIP J. Adv. Signal Process.* **2007**, 094386 (2007).

[30] Pavan, S., Schreier, R. & Temes, G. C. *Understanding Delta-Sigma Data Converters* (IEEE Press, Piscataway, 2017).

[31] Norsworthy, S. R. *Delta-Sigma Data Converters: Theory, design and simulation* (IEEE Press, New York, 1997).

[32] Mourjopoulos, J. Limitations of All-Digital, Networked Wireless, Adaptive Audio Systems, Audio Engineering Society 30th International Conference, 33 (2007).

[33] Melchior, V. High-resolution audio: a history and perspective. *J. Audio Eng. Soc.* **67**(5), 246–257 (2019).

[34] AN-1497 Filterless Class D Amplifiers, Application Report, Texas Instruments, Dallas (2013).

[35] Tatlas, N. A., Kontomichos, F. & Mourjopoulos, J. Design and performance of a sigma delta digital loudspeaker array prototype. *J. Audio Eng. Soc.* **57**(1), 38–45 (2009).

[36] Fujimori, I., Nogi, A. & Sugimoto, T. A multibit delta-sigma audio DAC with 120-dB dynamic range. *IEEE J. Solid State Circuits* **35**(8), 1066–1073 (2000).

[37] ISO 9613-1:1993, Acoustics—attenuation of sound during propagation outdoors—Part 1: Calculation of the absorption of sound by the atmosphere (1993).

[38] IEC 61260-1, Electroacoustics–octave-band and fractional-octave-band filters (2014).

[39] EdgeWave, Short Pulse Systems. Accessed July 20th 2020. https://www.edge-wave.de/web/en/produkte/short-pulse-systeme/.

[40] Class5Photonics, Products. Accessed July 20th 2020. https://www.class5photonics.com/products.html.

[41] Kaminow, I. P. *An Introduction to Electrooptic Devices* (Academic Press, New York, 1974).

[42] Pandey, P. K. & Thareja, R. K. Plume dynamics of laser produced air plasma. *J. Phys: Conf. Ser.* **208**, 012091 (2010).

[43] Rossi, M. *Acoustics and Electroacoustics* (Artech House, Norwood, MA, 1988).

[44] Audacity | Free, open source, cross-platform audio software. Accessed September 16th 2020. https://www.audacityteam.org/.

[45] B&O, Music for Archimedes. Copenhagen: Hansen and Munch (1991).

[46] Barnes, N. P. Solid-state lasers from an efficiency perspective. *J. Sel. Top. Quantum Electron.* **13**(3), 435 (2007).

