# Supplementary material for: Laser-sound: optoacoustic transduction from digital audio streams
Source: Sci Rep. 2021 Jan 22;11:476. doi: 10.1038/s41598-020-78990-z (PMC7822945; doi:10.1038/s41598-020-78990-z)
Supplement: Supplementary file 1 — Supplementary Material 1. [file 41598_2020_78990_MOESM1_ESM.pdf]

Supplementary information

Laser-Sound: optoacoustic transduction from digital audio streams

Konstantinos Kaleris[1,*], Björn Stelzner[2], Panagiotis Hatziantoniou[1], Dimosthenis Trimis[2], and John Mourjopoulos[1]

[1]University of Patras, Department of Electrical & Computer Engineering, Wire Communications Laboratory, Audio & Acoustic Technology Group, 26500 Rio, Greece.

[2]Karlsruhe Institute of Technology, Engler-Bunte-Institute, Karlsruhe, Germany.

Description of the audio files

The audio_listening_tests.rar file of the supplementary material contains the recordings (wavs) of:

- single sine waves with frequencies 125Hz, 250Hz and 500Hz
- sine sweep signal with frequency range 40Hz-1kHz

reproduced by the transducer prototype. Both 1-bit and 3-level ΣΔ modulation streams were used for comparison at laser repetition rates of 4kHz and 8kHz respectively. In addition, for the 1-bit ΣΔ, a short excerpt from pop-rock music was recorded and simulated via the model. The laser repetition rate for the music piece was 19.2kHz. It should be noted that, the differences between the simulated and recorded signals originated mainly in the ambient noise and acoustic distortions due to room reflections and reflections on the optical components, i.e, the optical table, the focusing lens etc. Moreover, at this high repetition rate, the energy of the laser pulses was around the breakdown threshold and for this reason, some of the pulses did not lead to breakdown. This effectively leads to an increase in the noise of the reproduced signal.

Moreover, the reproduction of music and female speech signals was simulated via the model assuming an ideal optoacoustic transducer based on a state-of-the-art laser system. Here, a 5-bit ΣΔ encoding and a laser repetition rate of 160kHz were assumed.

All signals were processed as described in the Signal Processing subsection of the Methods section. The acronym “LP” in the name of an audio file denotes Low-Pass Filtering for response equalization (see also subsection “Signal Processing”).

For the listening tests, the use of headphones is recommended.
